# Geographic and taxonomic notes, addenda and corrigenda on the subtribe Bembidiina Stephens, 1827 of the 2017 ‘Catalogue of Palaearctic Coleoptera’ (Coleoptera, Carabidae, Bembidiina)

**DOI:** 10.3897/zookeys.1044.62593

**Published:** 2021-06-16

**Authors:** Paolo Neri, Luca Toledano

**Affiliations:** 1 Via Guido Rossa 21 “San Lorenzo”, 47121 Forlì (FC), Italy Unaffilaited Forli Italy; 2 Museo Civico di Storia Naturale di Verona, Lungadige Porta Vittoria 9, 37129 Verona, Italy Museo Civico di Storia Naturale di Verona Verona Italy

**Keywords:** *
Asaphidion
*, *
Bembidion
*, *
Ocys
*, *
Sinechostictus
*, synonymy

## Abstract

Some corrections to the section of subtribe Bembidiina of the Catalogue of Palaearctic Coleoptera, Vol. 1, together with geographic, systematic, and synonymic updates are reported and commented upon. The following five new synonymies are proposed (with junior synonym listed first): Bembidion (Peryphanes) dostali Kirschenhofer, 1984 = Bembidion (Peryphanes) sanatum Bates, 1883 **syn. nov.**; Bembidion (Terminophanes) pseudoconsumatum Kirschenhofer, 1984 = Bembidion (Terminophanes) sjoelanderi Jedlička, 1965 **syn. nov.**; Bembidion (Asioperyphus) sapporense Jedlička, 1951 = Bembidion (Politophanes) chloreum Bates, 1873 **syn. nov.**; Bembidion (Peryphus) torosiense Jedlička, 1961 = Bembidion (Peryphus) subcostatum vau Netolitzky, 1913 **syn. nov.**; Sinechostictus (Sinechostictus) multisulcatus cariniger Korge, 1971 = Sinechostictus (Sinechostictus) multisulcatus (Reitter, 1890) **syn. nov**. Furthermore we confirm the following synonimies: *Asaphidion weiratheri* Netolitzky, 1935 = *Asaphidion ganglbaueri* J.Müller, 1921; Sinechostictus (Sinechostictus) effluviorum (Peyron, 1858) = Sinechostictus (Sinechostictus) tarsicus (Peyron, 1858). The following nine new combinations are proposed: Bembidion (Euperyphus) dimidiatum Ménétriés, 1832 **comb. nov.**; Bembidion (Peryphus) psuchrum Andrewes, 1922 **comb. nov.**; Bembidion (Plataphus) pseudolucillum Netolitzky, 1938 **comb. nov.**; Bembidion (Politophanes) chloreum Bates, 1873 **comb. nov.**; Bembidion (Politophanes) gotoense Habu, 1973 **comb. nov.**; Bembidion (Politophanes) shunichii Habu, 1973 **comb. nov.**; Bembidion (Politophanes) umeyai Habu, 1959 **comb. nov.**; Bembidion (Politophanes) yoshidai Morita, 2009 **comb. nov.**; Bembidion (Terminophanes) sjoelanderi Jedlička, 1965 **comb. nov.** The species *Bembidion psuchrum* Andrewes, 1922 and *Bembidion sanatum* Bates, 1883 are here redescribed.

## Introduction

After the publication of the Catalogue of Palaearctic Coleoptera we correct some mistakes found in the section of the Catalogue regarding the Carabidae, Bembidiina (Marggi et al. 2017) as identified during our studies or following the suggestions of colleagues. In this contribution we provide and discuss these corrections and add some systematic and geographic updates.

## Materials and methods

The systematic treatment of the Bembidiina and the geographic acronyms follow Löbl and Löbl (2017). The acronym YU includes the present Serbia (SB), Montenegro (ME), and Kosovo (KO). In the Current distribution sections, abbreviations are presented as they are in the catalogue. The abbreviations referring to new records are listed in bold. Body length was measured for card-mounted specimens from the front margin of the labrum to the apex of the elytra. The measurement of the aedeagus does not include the portion of endophallus protruding from the basal opening. Dissections were made using standard techniques. Genitalia and small parts were preserved in Euparal on acetate mounts fixed on the same pins as the specimens. The photographs of habitus were made by LT with Nikon DSFi1 and Nikon DS-L2 on a Leica Z6 microscope and those of the male genitalia by Gabriele Fiumi with a Nikon D300 on a Leitz Dialux 20 EB microscope. In the following text “the catalogue” refers to the section Bembidiina of the Catalogue of Palaearctic Coleoptera by Marggi et al. (2017).

The examined material is preserved in the following collections:

**AP** coll. Andreas Pütz, Eisenhuttenstadt, Germany;

**CR** coll. Christoph Reuter, Hamburg, Germany;

**CTVR** coll. Luca Toledano, Verona, Italy;

**DW** coll. David Wrase, Gusow Platkow (part of Zoologische Staatssammlung München, Germany);

**MHB** Museum für Naturkunde. Berlin, Germany;

**MSNT**Museo Civico di Storia Naturale, Trieste, Italy;

**NHMB**Naturhistorisches Museum, Basilea, Switzerland;

**NHMUK**Natural History Museum, London, England;

**NHMD** Natural History Museum, Copenhagen, Denmark;

**NHMW**Naturhistorisches Museum, Vienna, Austria;

**NHRS**Swedish Museum of Natural History, Stockholm, Sweden;

**NMPC**National Museum (Natural History), Prague, Czech Republic;

**PN** coll. Paolo Neri, Forlì, Italy;

**SMTD**Senckenberg Museum für Tierkunde, Dresden, Germany;

**VS** coll. Vladimir Skoupy, Kamenne Zhrovice, Czech Republic;

**VZ** coll. Vladimir Zieris, Pardubice, Czech Republic.

## Taxonomy

### Asaphidion ganglbaueri

Taxon classificationAnimaliaColeopteraCarabidae

J. Müller, 1921

E170B845-6646-55B2-B71F-0677F8108C2F

Asaphidion weiratheri Netolitzky, 1935

#### Material examined.

1 ♂, “Kysyldscha Hammam [= Kızılcahamam] / Paphlagonien, Asm. B. / Weirather, Innsbruck [printed] // *ganglbaueri* m. [handwritten] / det. J. Müller [printed]” (MSNT); 1 ♀, “Kysyldscha Hammam [= Kızılcahamam] / Paphlagonien, Asm. B. / Weirather, Innsbruck [printed]” (MSNT).

#### Notes.

*Asaphidion ganglbaueri* J. Müller, 1921 was described based on six specimens collected on Bosdagh, Turkey; it is very similar to *Asaphidion rossi* Schaum, 1857 but with the antennae completely blackish. *Asaphidion weiratheri* Netolitzky, 1935 was described from Turkey, and the type locality is Kysilka-Amam, near Ankara; the species is compared with *Asaphidion caraboides* Schrank, 1781 and its subspecies without any mentions of *A. ganglbaueri*. Müller (1937), examining topotypical specimens of *weiratheri* provided by Weirather himself and, after comparison with *ganglbaueri*, stated that they belonged to the same taxon. The synonymy was accepted by Lorenz (1998) and by Casale and Vigna-Taglianti (1999). Marggi et al. (2003) omitted to take into consideration the synonymy stated by Müller (1937) and listed *weiratheri* as a valid species; this treatment was later followed by Lorenz (2005) and Marggi et al. (2017). We requested loan of the type series of both species from NHMW but they are missing (Harry Schillhammer, pers. comm.).

#### Conclusions.

Considering that the topotypical specimens (Kysilka-Amam, Turkey) sent by Weirather and probably belonging to the type series were identified by Müller as *A. ganglbaueri*, that Netolitzky (1935) did not take into consideration or ignored the description of *A. ganglbaueri* when he described *A. weiratheri*, and that Müller (1937) himself stated the synonymy of both species after examination of both type series, we think that the synonymy stated by Müller (1937) should be retained until proven otherwise. Therefore we confirm that *Asaphidion weiratheri* Netolitzky, 1935 is a synonym of *Asaphidion ganglbaueri* J. Müller, 1921.

### Bembidion (Bembidion) quadripustulatum Audinet-Serville, 1821

We report the species from Syria: Syria, Dayr az Zawr, 10 km SE, Euphrates River bank (CTVR). Current distribution in Asia: **A**: CY, ES, FE. IN, IQ, IS, KI, KZ, LE, NMO, **SY**, TD, TM, TR, UZ, WS.

### Bembidion (Bembidionetolitzkya) atrocaeruleum Stephens, 1828

The mention for Austria provided by Degasperi et al. (2014) was not included in the catalogue. Current distribution: **E**: **AU**, BE, CZ, FR, GB, GE, IR, IT, NL, PT, SK, SP, SZ, UK.

### Bembidion (Bembidionetolitzkya) rhodopense Apfelbeck, 1902

The mention for Greece by Mūller-Motzfeld and Marggi (2011) was not reported in the catalogue. Furthermore we report the species for Montenegro: Montenegro, Plav, Vusanje, torr. Grija, 1000 m (PN). Current distribution: **E**: AL, BU, **GR**, MC, **ME**, YU.

### Bembidion (Emphanes) netolitzkyanum Schatzmayr, 1940

We mention the species for Syria. Syria, 20 km S of Kassab (PN); Syria, Al Querdaha (VS).

Present distribution: **A**: IS, **SY**.

### Bembidion (Euperyphus) dimidiatum

Taxon classificationAnimaliaColeopteraCarabidae

Ménétriés, 1832 comb. nov.

1F126190-BC8E-593D-9B82-A03FD8AD3328

Bembidion (*incertae sedis*) dimidiatum Mènètriès, 1832

#### Notes.

Mènètriès (1832) describes Bembidion (Peryphus) dimidiatum from the banks of “Potkoumà” near “Pétigorsk” on “quelques individus”. After the description, *B. dimidiatum* was retained as strictly related to *B. oblongum* Dejean, 1831 or *B. ripicola* Dufour, 1820 (currently all species belonging to the subgenus Euperyphus Jeannel, 1941). Netolitzky (1914a) synonymized *B. dimidiatum* with *B. tricolor* (Fabricius, 1801) (= *B. varicolor* (Fabricius, 1803); the synonymy derives from the fact that the author examined Caucasian specimens identified as *B. dimidiatum* actually belonging to B. (Bembidionetolitzkya) varicolor and was perhaps unaware that this last species also occurs in the Caucasus and shares the same particular pattern of color of the European *varicolor*. But later (1935) the author himself retained *B. dimidiatum* as closely related to *oblongum*, and in any case not similar to *B. conforme* Dejean, 1831 or *B. tricolor* (both belonging to subgenus Bembidionetolitzkya Strand, 1929). Netolitzky (1943a: 112/84, note 83) reported that *B. dimidiatum* belongs to the “Gruppe des *B. ripicola* – *oblongum*” and retained it as close to *B. testaceum parallelipenne* Chaudoir, 1850; moreover, Netolitzky (1943b: 17/113, note 18) mentioned that, if they were synonyms, *B. dimidiatum* should have priority over *B. parallelipenne*; he did not formally state in any way this synonymy. Later, Kryzhanovskij et al. (1995) and Lorenz (1998) listed *B. dimidiatum* as a synonym of *B. parallelipenne* but expressed doubts. Marggi et al. (2003: 271) completely changed direction, moving *dimidiatum* to “incertae sedis”, explaining the decision as follows: “Bembidion (Peryphus) parallelipenne ? syn. *dimidiatum* Ménétriés, 1832, resurrected as *Bembidion* (incertae sedis) *dimidiatum* Ménétriés, 1832. It is not a member of *Actedium*, as given in Lorenz, 1998” (Marggi et al. 2003: 21).

The same settlement is confirmed by Lorenz (2005) and Marggi et al. (2017). Unfortunately, the sentence of Marggi et al. (2003) above and in particular the reference to *Actedium* probably caused the moving of *B. dimidiatum* to “incertae sedis”, and are unclear; we verified that in Lorenz (1998) neither *B. parallelipenne*, nor *B. dimidiatum* are reported as belonging to the subgenus Actedium. From the literature we ascertained that *B. dimidiatum* certainly belongs to the subgenus Euperyphus (as currently intended, see above); there still remains uncertainty regarding its precise position, with three (*B. parallelipenne parallelipenne*, *B. parallelipenne exisonum* Lutshnik, 1938, *B. parallelipenne pseudoripicola* Iablokoff-Khnzorian, 1963) taxa currently assigned to the same group. In our opinion it seems unlikely that all the three Caucasian forms could be valid, and it would be necessary to verify them; in any case we suppose that the subspecies more similar to *B. dimidiatum*, mainly due to the elytral coloration, could be *B. pseudoripicola*; in case of possible synonymies the problem of priority already reported by Netolitzky (1943b: 17/113, note 18) mentioned above should be considered. However *B. dimidiatum* must be included in the subgenus Euperyphus as currently intended.

### Bembidion (Neja) ambiguum Dejean, 1831

*Bembidion hesperus* Crotch, 1867 (Lindroth 1960) must be added to the list of synonyms of *B. ambiguum* Dejean, 1831.

### Bembidion (Ocydromus) hiekei Müller-Motzfeld, 1986

We report the species for Afghanistan: Afghanistan, Cheikehabad (CTVR). Current distribution: **A**: **AF**, IN, TM.

### Bembidion (Ocydromus) semilotum Netolitzky, 1911

We report the species from Iraq: N Iraq, W Kurdistan, SE Al-Amādīya (DW, CTVR). Current distribution: **E**: AR; **A**: IN, **IQ**, TR.

### Bembidion (Ocydromus) siculum durudense Marggi & Huber, 1999

We report the species from Iraq: N Iraq, N Choman, Halgurd Mt., 3200–3400 m (CR). Current distribution: **E**: AB, AR, GG, ST; **A**: IN, **IQ**, TR.

### Bembidion (Ocydromus) toledanoianum Echaroux, 2008

We report the species for Turkey: Riviera bei Kemer, M5/96, Sieber (DW) and Prov. Mugla, north Dalaman (CTVR). The species was formerly known only from Rhodes, Greece. Current distribution: **E**: GR (Rhodes); **A**: **TR**.

### Bembidion (Ocyturanes) kurdistanicum Netolitzky, 1920

We report the species from Iraq: N Choman, Halgurd Mt., 3200–3400 m (PN, CTVR). Current distribution: **A**: TR, IN, **IQ**.

### Bembidion (Ocyturanes) praeustum Dejean, 1831

We report the species from Iraq: Rawandoz, Akolan valley, 1900–2000 m (PN). Current distribution: **E**: AL, BH, BU, CR, FR, GR, IT, MA, MC, RO, SB, SL, ST, TR, UK, YU; **N**: EG, LB; **A**: CY, IN, **IQ**, IS, JO, LE, TR.

### Bembidion (Ocyturanes) ronfelixi Neri & Toledano, 2017

We report the species from Tajikistan: Pamir-Alai, Seravahan-Mts., Zavron Valley, 2100–3000 m (DW, PN). Current distribution: **A**: KI, KZ, **TD**, TM, UZ.

### Bembidion (Odontium) striatum (Fabricius, 1792)

We report the species from Greece: Macedonia, Lake Kerkini c/o Megalochori (CTVR, PN) and Sidirokastro (CTVR); from Poland: 5 Km NE Lezajsk, Kurilowica env., banks San River (CTVR) and Kozki, Bug River (CTVR, VZ); from Kazakhstan: KZ or., Kurkum distr., 50 km N Zaisan, Chernyi river, 400 m (CTVR, AP). Current distribution: **E**: AL, AU, BH, BU, BY, CR, CT, CZ, DE, EN, FI, FR, GE, **GR**, HU, IT, LA, LT, MC, MD, NL, NT, **PL**, PT, RO, SK, SL, SP, ST, SZ, TR, UK, YU; **A**: **KZ**, TR, WS.

### Bembidion (Omotaphus) mixtum Schaum, 1863

In the revision of the subgenus Omotaphus Netolitzky, 1914 Bonavita et al. (2016) stated that both *B. madagascariense* Chaudoir, 1876 and *B. picturatum* Fairmaire, 1898 were valid species and not synonyms of *B. mixtum* Schaum, 1863. They also stated that *B. stricticolle* Jeannel, 1955 was a synonym of *B. madagascariense*, and that *B. tumidum* Gemminger & Harold, 1868 and *B. variegatum* Boheman, 1848 were synonyms of *B. mellissi* Wollaston, 1869; all of these African species were formerly listed as synonyms of *B. mixtum* and therefore they must be removed from the list of synonyms of *B. mixtum*.

### Bembidion (Pekinium) chinense Csiki, 1928

Bembidion (Pekinium) chinense Csiki, 1928, type species of the monospecific Bembidion subgenus Pekinium Csiki, 1901, was declared “species inquirenda” by Toledano and Sciaky (1998); this species must be transferred to the group of species “incertae sedis”.

### Bembidion (Peryphanes) dalmatinum dalmatinum Dejean, 1831 and Bembidion (Peryphanes) dalmatinum levantinum Všetečka, 1941

Bembidion (Peryphus) dalmatinum levantinum Všetečka, 1941, was described from Syria (Damascus), Lebanon (Chtaura, Hamana) and Palestine (Haifa, today Israel); the description is in the keys for the identification of the subspecies of *B. dalmatinum* Dejean, 1831 (Všetečka 1941); the diagnostic character mentioned for *levantinum* is the color of the femora, completely reddish yellow. Netolitzky (1943b) includes Cyprus in the distribution of *B. levantinum* after examination of specimens of the Splichal-Spiller collection. Jeanne (1986) mentioned *B. levantinum*, assigned to the subgenus Peryphanes, from several localities in Cyprus. Austin et al. (2008) also mentioned the species for Cyprus. Marggi et al. (2017) mentioned *B. dalmatinum levantinum* for Lebanon, Israel, and Cyprus. We received from NHMW the specimens from Cyprus of the Splichal-Spiller collection seen by Netolitzky and we ascertained that the femora actually are blackish at base or on the lower side. We could also examine some specimens from Cyprus (SMTD, MHB, PN, SDEI) and all of them except for a few immature specimens have femora more or less darkened at the base or on the lower side.

Since the difference between *B. dalmatinum levantinum* and *B. dalmatinum dalmatinum* is only in the darkening of the base of the femora, we suppose that only the nominotypical form is present in Cyprus: therefore Cyprus must be removed from the distribution of the subspecies, while Syria must be added, according to the original description. Current distribution of *B. levantinum*: A: IS, LE, SY. On the other hand Cyprus must be added to the distribution of *B. dalmatinum dalmatinum*.

### Bembidion (Peryphanes) deletum Audinette-Serville, 1821

The nominotypical form of *B. deletum* Audinette-Serville, 1821 is not present in Spain, as already (correctly) supposed by Serrano (2003) and Ortuño and Toribio (2005). All records from Spain must be referred to Bembidion (Peryphanes) deletum schulerianum Müller-Motzfeld, 1986 and Spain must be removed from the distribution of *B. deletum deletum*.

### Bembidion (Peryphanes) fraxator

Taxon classificationAnimaliaColeopteraCarabidae

Ménétriés, 1832

E2731ED8-2B6A-5E2A-9FA5-0B24B4536B3F

Bembidion (Peryphanes) lucidum (Faldermann, 1836)

#### Notes.

*Bembidion lucidum* Faldermann, 1836 was retained, after a study of the type specimen by Netolitzky (1910) as being very close to *B. dalmatinum* Dejean, 1831. Later Netolitzky (1914b) synonymized *lucidum* with *dalmatinumfraxator* Ménétriés, 1832 (currently *fraxator* is considered a valid species). Marggi et al. (2003: 271) listed *B. lucidum* as a synonym of *B. dimidiatum* Ménétriés, 1832 which was moved to “incertae sedis”; while the moving of *B. dimidiatum* was mentioned (p. 21) nothing was stated about *B. lucidum*. This situation remains also in Marggi et al. (2017).

After having carefully analyzed the literature regarding these taxa (e.g., Ménétriés 1832; Faldermann 1836; Schaum 1861; Netolitzky 1910, 1914b; Kryzhanovskij et al. 1995; Belousov and Sokolov 1996), we confirm that *B. lucidum* Faldermann is a synonym of *B. fraxator* Ménétriés and not of *B. dimidiatum* Ménétriés, which, moreover, belongs to another subgenus (see above).

### Bembidion (Peryphanes) sanatum

Taxon classificationAnimaliaColeopteraCarabidae

Bates, 1883

2098A5DB-6D88-56C8-B8C7-6936F4207AD6

Figures 1 , 5 , 6

Bembidion (Peryphanes) dostali Kirschenhofer, 1984 syn. nov.

#### Material examined.

1 ♂, holotype of Bembidion (Peryphus) sanatum Bates, “Type [round, bordered in red] // Japan / G. Lewis / 1910 – 310 [printed] // *B.* / *sanatum* / Bates [handwritten]” (NHMUK); 1 ♂, “Museum Paris / Nippon Moyen / E. Gallois 1912 [printed] // Japon. Chu- / zenji 17.8.1909 / Edme Gallois [yellow, printed] // coll. Netolitzky [printed] // *sanatum* Bts / dt Netolitzky 1937 [handwritten] // Coll. Netolitzky [printed]” (NHMW); 2 ♂♂, 3 ♀♀, paratypes of *B. dostali*, (NHMW, CTVR), with the same labels as the holotype of *B. dostali*.

The type specimen of *B. sanatum* is rather well preserved, except for the broken antennae (the left one complete but divided in three portions, the right one missing three antennomeres in the middle) and legs (the right median leg and the hind right leg with detached tarsi). All the broken parts are glued on the label.

The specimen from NHMW, identified as *sanatum* by Netolitzky and also used by Kirschenhofer (1984) in the description of Bembidion (Peryphus) dostali (at present assigned to the subgenus Peryphanes Jeannel, 1941), as an example of the closest species, is in very bad condition: completely immature, missing almost all the appendages of the head and a part of the legs; the pronotum is evidently flattened, the elytra are open and the specimen is so immature that a dissection of the male genitalia (Kirschenhofer, 1984 mistakenly mentions this specimen as a ♀) is not recommended. The general habitus and the comparison with the type confirmed the determination of Netolitzky.

Four of the paratypes of *B. dostali* from NHMW are immature. The only mature male, originally glued to the label on the dorsal side, has been dissected by us and mounted again in order to leave the dorsal side visible; the specimen lacks the three left legs.

We also examined photographs of the holotype of Bembidion (Peryphus) dostali Kirschenhofer, 1984 kindly provided by Alexey Solodovnikov and Mikkel Høegh Post (NHMD), and of its labels: “Japan, Unzen / 32°46’N, 130°16’E / 19.VII.1934 / Eigin Suenson leg. [printed] // Holotypus [red, handwritten] // *Bembidion* [printed] / *dostali* n.sp. [handwritten] / det.: Kirschenhofer [printed] 82 [handwritten]”.

#### Type locality.

Niohozan [Giappone], “in June (…) near the snow” (Bates 1883).

#### Redescription of the holotype

**Figures 1–4.**
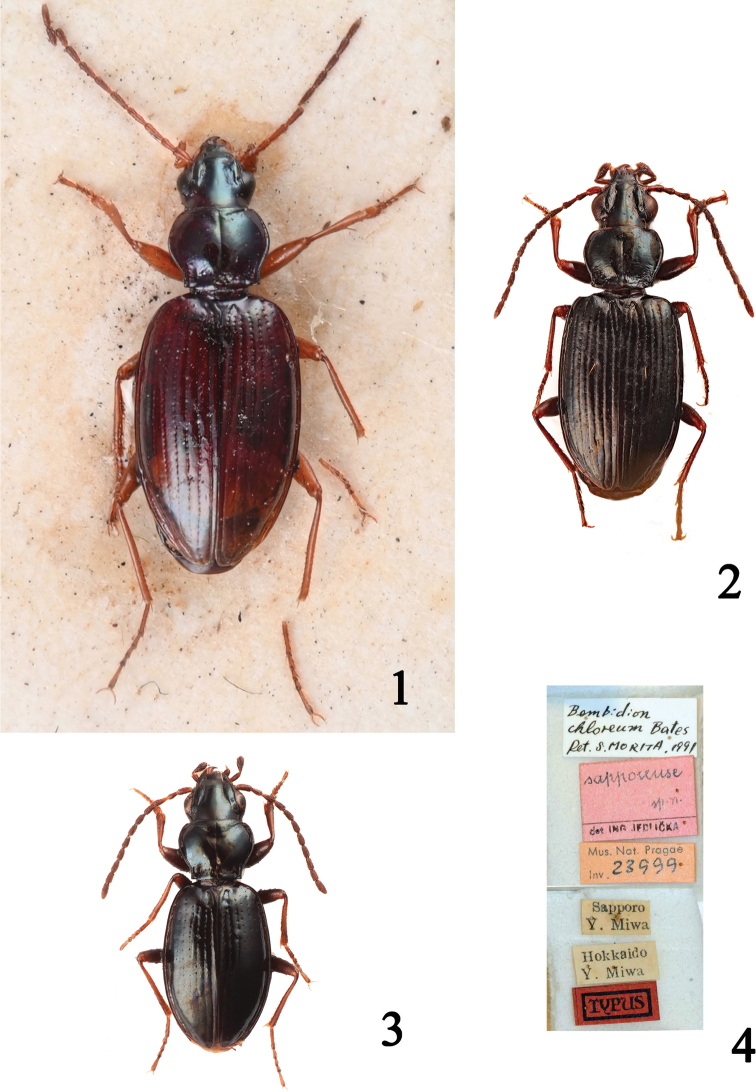
Habitus of **1**Bembidion (Peryphanes) sanatum Bates, 1883, holotype (NHMUK), 5.60 mm **2**B. (Plataphus) pseudolucillum Netolitzky, 1938 from Kibune, Kyoto (NHMW), 4.20 mm **3**B. (Peryphus) psuchrum Andrewes, 1935 Inde, Himachal Pradesh, Mahari, 3500 m (CTVR), 3.50 mm **4** labels of the holotype of *Bembidion sapporense* Jedlička (NMPC). Photographs: Luca Toledano.

♂ (Fig. 1). Total body length 5.60 mm. ***Coloration***: head and pronotum dark brown; elytra brownish olive. Legs and palps orange. Antennae orange, slightly darkened from fifth antennomere. ***Head***: maximum width, including eyes, 1.02 mm; interocular distance 0.63 mm; frons, clypeus, and neck smooth; evident frontal furrows ending posteriorly between the first and the second supraorbital seta. Eyes weakly protruding, temples oblique towards the neck. Antennae long 3.02 mm. ***Pronotum***: length along the midline 1.06 mm; width of anterior margin 0.91 mm, maximum width 1.31 mm, width of base 0.98 mm; pronotal width/pronotal length ratio 1.23; convex, more evidently near the anterior angles; sides entirely rebordered, narrowing with evident sinuate shape before the base, with which they form a slightly acute corner; lateral gutter narrow, of homogeneous width; complete surface glossy; laterobasal carina very long and evident; median line sharp, transverse anterior semilunar impression more evident; basal transverse impression punctured between the deep basal foveae. ***Elytra***: length 3.55 mm, maximum overall width, at middle of elytra, 2.25 mm; oval, shoulders slightly rounded; humeral margin reaching stria 5; full microsculpture, so fine and irregularly transverse that it looks like shagreening. Striae with punctures clearly visible almost to apex, even though less impressed at apex.

**Figures 5–9.**
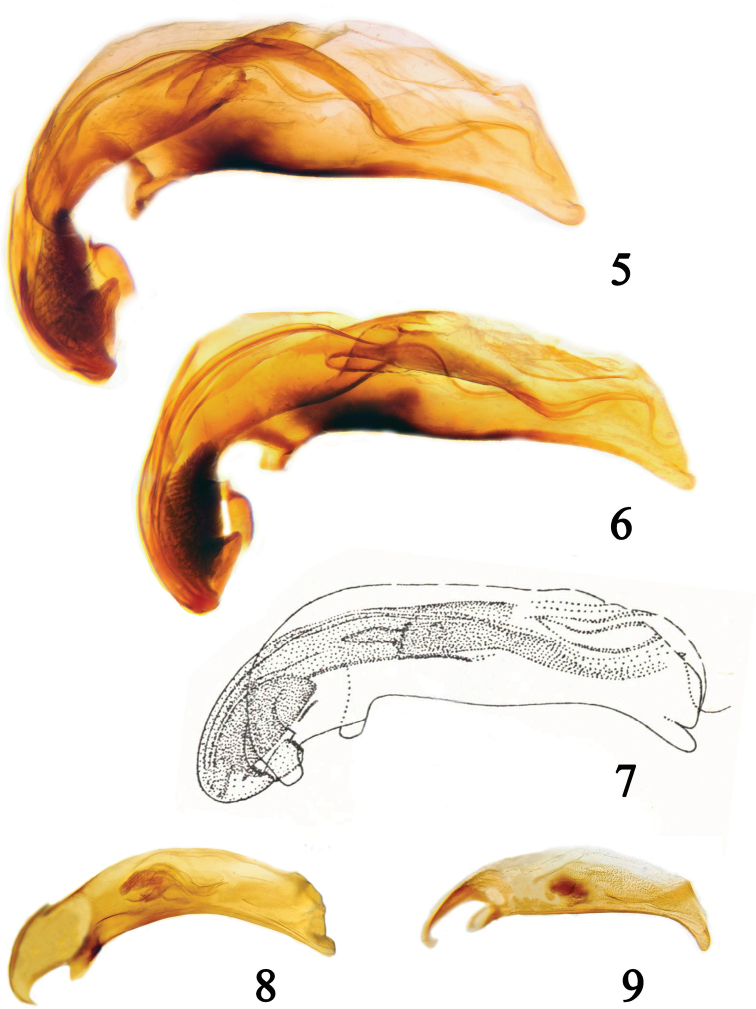
Median lobe of the aedeagi of **5**Bembidion (Peryphanes) sanatum Bates, 1883, holotype (NHMUK), 1.55 mm **6**B. (P.) dostali Kirschenhofer, 1984, paratype (NHMW), 1.53 mm **7**B. (P.) lulinense Habu, 1973 (drawing from Habu 1973), 1.41 mm **8**Bembidion (Peryphus) psuchrum Andrewes, 1935, holotype (NHMUK), 0.91 mm **9**Bembidion (Plataphus) pseudolucillum Netolitzky, paratype (NHMW), 0.80 mm.

***Male genitalia*.** Aedeagus (Fig. 5) of medium-large size (1.55 mm), ventral margin with a very faint gibbosity and apex only slightly bent ventrally; central brush completely protruding from basal opening; paracopulatrix lamina and main sclerite extending towards the apex, paracopulatrix lamina narrowing anteriorly; parameres of same length (terminology according to Neri and Vigna Taglianti 2010).

#### Conclusions.

The examination of the holotype of *B. sanatum*, currently listed as *Bembidion* “incertae sedis” in Marggi et al. (2017), shows that the species must be assigned to the subgenus Peryphanes Jeannel, 1941, as correctly stated by Sundukov and Makarov (2016), unfortunately unavailable to us before the publication of the catalogue in 2017. The examination of the material of *B. dostali* (Fig. 6) instead revealed that the taxon, formerly considered closely related to *B. sanatum*, is conspecific. The differences between the pronota, mentioned and drawn by Kirschenhofer (1984: 91), are due to the comparison with a completely immature specimen of *B. sanatum* (examined by us, see above); furthermore the examined paratypes of *B. dostali* show a pronotum with characters that are slightly variable. The aedeagi are very similar to one another (Figs 5, 6). According to these observations we can state that Bembidion (Peryphanes) dostali Kirschenhofer, 1984 = Bembidion (Peryphanes) sanatum Bates, 1883 syn. nov. We added the following label to the examined specimens: Bembidion (Peryphanes) sanatum Bates – det. Neri and Toledano 2020.

*Bembidion sanatum*, a Japanese species, is distinguished from the related species of Japan and China by the following characters: from *B. hykosanum* (Habu & Ueno, 1955) (JA) and *B. parepum* Jedlicka, 1933 (SCH) by the completely microsculptured or shagreened elytra, from *B. hayachinense* (Nakane, 1979) (JA) by the first three antennomeres light and the pronotum not microsculptured, and from *B. lulinense* Habu, 1973 (TAI) by the structure of the endophallus (Fig. 7).

### Bembidion (Peryphus) asiaeminoris Netolitzky, 1935

In Mandl (1963)Bembidion (Peryphus) asiaeminoris Netolitzky, 1935 is reported from “Iran (Kuh-räng, östl. Isfahan)”; its presence in Iran is reported also by Marggi et al. (2017). We received two females of the three specimens mentioned by Mandl from NHMB: the examination of these specimens led to a change of identification, revealing that they belong to Bembidion (Peryphanes) morvanianum Mūller-Motzfeld, 1986 (= *Peryphus zagrosensis* Morvan, 1973). We are unaware of any other records of *B. asiaeminoris* from Iran, therefore Iran must be removed from the distribution of *B. asiaeminoris*. Current distribution: **E**: AB, AR, ST; **A**: TR.

### Bembidion (Peryphus) charon Andrewes, 1926

In the catalogue the species is reported from Japan, but we think that it is likely to be a mistake, as we are unaware of any reports of the species from this area: therefore Japan must be removed from the distribution of the species. Current distribution: **A**: NP, UP.

### Bembidion (Peryphus) cordicolle du Val, 1852

The mention for Bulgaria provided by Hieke and Wrase (1988) was omitted in the catalogue. Current distribution: **E**: **BU**, GR; **A**: TR.

### Bembidion (Peryphus) ehikoense Habu, 1984

The species, described from Japan and originally assigned to the subgenus Peryphus Dejean, 1821, was subsequently listed as “incertae sedis” (e.g., Marggi et al. 2003; Lorenz 2005; Marggi et al. 2017). The study of the original description and of the aedeagus confirm that the author correctly assigned the species to *Peryphus*.

### Bembidion (Peryphus) kuhitangi Mikhailov & Belousov, 1991

We noticed that the species name in the catalogue is reported on page 339 with the incorrect spelling “*kughitangi*” (sic!) and must be replaced with “*kuhitangi*”; furthermore, the species has been transferred from “incertae sedis” to the subgenus Peryphus Dejean, 1821 by Neri and Toledano (2017).

### Bembidion (Peryphus) obscurellum

Taxon classificationAnimaliaColeopteraCarabidae

Motschulsky, 1845

389DE621-EB16-5A8A-AAAB-A928EACB2C33

Bembidion (Peryphus) obscurellum turanicum Csiki, 1928
Bembidion (Peryphus) obscurellum fumipenne Fassati, 1957
Bembidion (Peryphus) obscurellum thibeticum Fassati, 1957
Bembidion (Peryphus) obscurellum insperatum Lutshnik, 1938

#### Notes.

Fassati (1957) describes Bembidion (Peryphus) fuscicrum thibeticum ssp. nov. and Bembidion (Peryphus) fuscicrum fumipenne nat. nov.; they are distinguished from the nominotypical form only by the coloration of the elytra. Lindroth (1963) synonymizes the following taxa with *B. obscurellum* Motschulsky, 1845: *B. fuscicrum* Motschulsky, 1855, *B. fuscicrum turanicum* Csiki, 1928, *B. fumipenne* and *B. thibeticum*, the last two taxa because they are only color varieties. Kryzhanovskij et al. (1995) listed *B. turanicum* as a subspecies of *B. obscurellum* and retained *B. fumipenne* and *B. thibeticum* as varieties only; moreover Belousov’s note (Kryzhanovskij et al. 1995: 85, note no. 158) reports that the subspecific situation of this polymorphic species is provisional. Recently, some authors (e.g., Lorenz 2005; Marggi et al. 2017) still list *B. turanicum*, *B. fumipenne*, and *B. thibeticum* as subspecies of *B. obscurellum*.

In light of our studies we maintain that the synonymies proposed by Lindroth (1963) were correct; moreover, our colleague Liang Hongbin (IZCAS, Beijing) confirms our opinion based on the study of hundreds of specimens from Mongolia that clearly show the variability of the species (Liang Hongbin, pers. comm.).

Regarding Bembidion (Peryphus) obscurellum insperatum Lutshnik, 1938, since its discriminating characters are not exclusive but shared by other subspecies, we evaluated the synonymy expressed in Marggi et al. (2017) and add to the distribution of the species the Russian Caucasus (ST), the region mentioned in the description, as already reported in Kryzhanovskij et al. (1995): Ciscaucasia, Western and Central Greater Caucasus. Current distribution: **E**: DE, NT, **ST**, SV; **A**: AF, CH, ES, IN, KA, KI, KZ, MG, TM, TR, WS, XIZ; **NAR**.

### Bembidion (Peryphus) psuchrum

Taxon classificationAnimaliaColeopteraCarabidae

Andrewes, 1922 comb. nov.

26ED701B-0AD4-5AD0-8CE6-F9251E75751A

Figures 3 , 8

Bembidion (*incertae sedis*) psuchrum Andrewes, 1922

#### Examined material.

1 ♂, holotype of *Bembidium psuchrum* Andrewes, “♂ 1995 [no. dissection by Müller-Motzfeld] // Holo / type [round, bordered in red] // Type [round, bordered in red] // *Bembidium / psuchrum* Andr. / Type [handwritten] / H. E. Andrewes det. [printed] // Brit. Mus. / 1923-24 // Bunderdhunga V. / W. Almora Divis. / 8000–12000 feet / June’ 19 H.G.C. [printed] // 3120 [printed]” (NHMUK); 1 ♂, “Inde / Himachal Pradesh / Mahari // 3500m / 18.VIII.80 / G. Ledoux” (CTVR) (Fig. 3).

#### Note.

The holotype is in perfect condition and the slide with the aedeagus, on a label mounted on the same pin as the specimen, is clear and well preserved.

#### Type locality.

Kumaon, West Almora, Bunderdhunga, 8000–12000 ft. [India, Uttarakhand] (Andrewes 1922).

#### Redescription of the holotype.

Total body length 3.90 mm. ***Coloration***: head, pronotum and elytra black, metallic. Palps dark brown with last palpomere light. Legs with femora black metallic and apex slightly lighter, tibiae reddish and tarsi reddish with blackish metallic reflections. Antennae reddish black with metallic reflections. ***Head***: maximum width, including eyes, 0.82 mm; interocular distance 0.55 mm; frons and clypeus smooth with a few transverse lines, neck microsculptured at base; evident, smooth, deep frontal furrows, ending behind at the second supraorbital seta. Eyes moderately convex, temples slightly convex and oblique towards neck. Antennae long 2.05 mm. ***Pronotum***: length along mid line 0.84 mm; width of anterior margin 0.77 mm, maximum width 0.93 mm, width of base 0.61 mm; pronotal width/pronotal length ratio 1.11; very convex, barely transverse; sides entirely rebordered, narrowing with an evident sinuous curve towards the base with which they form a slightly acute corner; marginal gutter very narrow, of homogeneous width; whole surface glossy, laterobasal carina not very evident; median line evident, slightly widened at base; transverse anterior semilunar impression with a few punctures; basal transverse impression punctured between the subquadrate, not deep, basal foveae. ***Elytra***: length 2.37 mm, maximum overall elytral width, slightly beyond the middle, 1.59 mm; oval, moderately convex, evident and rounded shoulders; humeral margin reaching stria 5; totally glossy, faint trace of microsculpture only at the extreme apex. Striae with evident punctation, mainly on disc, barely visible at apex; the very faint punctation is visible also at apex. Evident apical stria.

#### Male genitalia.

Medium sized (0.91 mm) aedeagus (Fig. 8), concave ventral margin with apex bent ventrally; central brush completely inside the median lobe, medium sized main sclerite, moderately wide and sinuate.

#### Conclusion.

The study of the holotype of the species, currently listed as *Bembidion* “incertae sedis” (Marggi et al. 2017), suggests that the species could be assigned to the subgenus Peryphus Dejean, 1821.

### Bembidion (Peryphus) subcostatumvau

Taxon classificationAnimaliaColeopteraCarabidae

Netolitzky, 1913

8C0A9448-8AE3-5274-A5A6-063219FD9F85

Bembidion (Peryphus) torosiense Jedlička, 1961 syn. nov.

#### Material examined.

***Holotype*** ♀ of Bembidion (Peryphus) torosiense Jedlička, 1961, “Anatolia – Toros / Berendi Eregli / 2000m, leg. Muche [printed] // Staatl. Museum für / Tierkunde Dresden [yellow, printed] // Holotypus [red, printed] // Ankauf Muche [yellow, printed] // *Bembidion* / *Peryphus* / *torosiense* sp.n. [pink, handwritten] / det. ING. JEDLICKA [pink, printed] // = *Bembidion* / *subcostatum vau* Net. / det. P. Bonavita, 2012 [printed]” (SMTD).

#### Notes.

In Marggi et al. (2017) the species is listed with the *Bembidion*, species “incertae sedis”. The comparison of the holotype with specimens of *B. subcostatum vau* Netolitzky, 1913 from the same locality, Eregly, Turkey (PN, CTVR) confirmed the synonymy noticed by Bonavita but never published. We wish to thank our colleague Paolo Bonavita for reporting this synonymy and allowing us to publish it here.

### Bembidion (Philochthus) judaicum Sahlberg, 1908

We report the species from Iran: Chahar Mahail va Bachtiari, Zagros Mts, 5 Km SW Asad Abad, 2400 m (DW). Current distribution: **E**: AB, BU, GG, GR; **A**: **IN**, IQ, IS, SY, TR.

### Bembidion (Philochthus) pallidiveste Carret, 1905

We report the species from Kazakhstan: Esil (CTVR). Current distribution: **E**: MD, ST, UK; **A**: CT, IN, IQ, IS, **KZ**, SY, TR.

### Bembidion (Plataphus) prasinum Duftschmid, 1812

We report the species from Montenegro: Mont., Zaton, V.76, leg. Sama (PN), first mention for the balcanic-mediterranean region. Current distribution: **E**: AU, BE, CZ, FI, FR, GB, GE, HU, IT, **ME**, NL, NR, NT, SK, SL, SP, SV, SZ, UK; **A**: ES, FE, KZ, MG, WS.

### Bembidion (Plataphus) pseudolucillum

Taxon classificationAnimaliaColeopteraCarabidae

Netolitzky, 1938 comb. nov.

72A96E57-2D16-5EF9-862C-6B110F5D5552

Figures 2 , 9

Bembidion (Peryphus) pseudolucillum Netolitzky, 1938

#### Material examined.

1 ♀, “Japan / Settsu / Katsuoji / 16.VI.24 / J.E.A. Lewis [printed] // 199 [handwritten] // Type [red, printed] // *pseudolucillum* Net. / Type Netolitzky 37 // *Peryphus* // H.E. Andrewes coll. / B.M. 1945-97 [printed]” (NHMUK). 3 ♀♀, on the same pin, “Oku Nikko / Nabagawa / 8.9.1937 / Jano, Jap. [handwritten] // *pseudolucillum* / det. Netolitzky [handwritten] // H.E. Andrewes coll. / B.M. 1945-97 [printed]” (NHMUK). 1 ♀, “Japan / Settsu / Katsuoji / J.E.A. Lewis [handwritten] // [one deleted label] // *Peryphus* sp. / striis mediocr- / punctat [illegible] / dt Netolitzky [handwritten] // mit Type / Brit. Museum / identisch Net. [handwritten] // Coll. / Netolitzky [printed] // *pseudolucillum* Net / Cotypus Netolitzky 37 [handwritten] // CO / TYPUS [red, printed] // Coll. / Netolitzky [printed]” (NHMW). 1 ♂, “Oku Nikko / Nabagawa / 8.9.37 Jano [handwritten] // *pseudolucillum* / dt. Netolitzky [handwritten, contoured in red] // coll. / Netolitzky [printed]” (NHMW); from the handwritten label contoured in red we deduce that this last specimen was considered by Netolitzky as a paratype. 1 ♂, 2 ♀♀, on the same pin, “Oku Nikko / Nabagawa / 8.9.37 Jano [handwritten] // coll. / Netolitzky [printed] // *pseudolucillum* / dt. Netolitzky [handwritten]” (NHMW); we mounted the ♂ on a separate pin, the labels have been photocopied in order to mount the specimens on separate pins, and the ♂, dissected, bears the slide with the aedeagus mounted in euparal on the same pin. 1 ♂, “Kibune, Kyoto / 12-IV-1950 / Leg. S. Uéno [handwritten] // Acqu.-Nr. / 1951-23 [printed] // Coll. / Meyer // *pseudolucillum* / Netolitzky 1938 [handwritten] / Det. S. Uéno [printed]” (NHMW) (Fig. 2). 2 ♀♀, “Kibune, Kyoto / 12-IV-1950 / Leg. S. Uéno [handwritten] // Acqu.-Nr. / 1951-23 [printed] // Coll. / Meyer // *pseudolucillum* [handwritten] / det. Paul Meyer [printed]” (NHMW). 1 ♂, “Japan G. Lewis // Japan. Lewis // Sharp Coll. 1905-313 // *Bembidion* cfr. *pseudolucillum* det. L. Toledano 2008 // CTVR Coll. Toledano Verona – Italy” (CTVR). 1 ♂, “Kujukawa / Aomori Pref. / Honehu Japan / 10.9.54 [handwritten] // Bembidion / pseudolucillum / Netolitzky 1938 / det. L. Toledano 2011 [printed] // CTVR / Coll. Toledano / Verona – Italy [printed]” (CTVR). 7 ♀♀, “Numata, Yumma Pref., Honshu Japan” (CTVR, PN). 1 ♀, “Amagi-Pass, Kawazu-cho, Shizuoka pref.” (CTVR). 1 ♀, “Sanjo-valley, Tabayama vill., Yamanashi-pref.” (CTVR). 1 ♀, “J. Honshu, Shiga-ken, Mikunidakeyama, E slope, 700 m” (CTVR). 2 ♀♀, 3 ♂♂, “Japan, Mt. Hiba, Saljo, Hiroshima pref.” (CTVR, PN).

#### Notes.

Netolitzky (1938) describes Bembidion (Peryphus) pseudolucillum from Settsu (Japan), assigning it to the group of *B. nitidulum* Marsham, 1802, s. l. (= *B. deletum* Serville, 1821); again Netolitzky (1943a, 1943b) found it difficult to find a correct position for the species in his Bestimmungs-Tabellen: he mentions similarities both with species of two subgenera, *Bembidionetolitzya* Strand, 1929 and *Peryphus* Dejean, 1821, but preferred the latter. Jedlička (1965), in his monograph on the Bembidiini of Eastern Asia, includes the species in the keys for subgenus Plataphus Motschulsky, 1864 and that of the subgenus Peryphus. The species is listed in “incertae sedis” in the last catalogues (e.g., Marggi et al. 2003; Lorenz 2005; Marggi et al. 2017).

#### Conclusions.

The examination of the holotype and of the aedeagus (Fig. 9) shows that the species belongs to subgenus Plataphus Motschulsky, 1864. The doubts regarding the systematic position of the species are due to its pronotal convexity, more evident than in other species of the subgenus. We added to all the non-type specimens the following label: Bembidion (Plataphus) pseudolucillum Net. – det. Neri and Toledano 2020. The species length varies from 3.6 to 4.7 mm, the aedeagus from 0.75 to 0.84 mm.

### Bembidion (Politophanes) chloreum

Taxon classificationAnimaliaColeopteraCarabidae

Bates, 1873 comb. nov.

A9636FD2-9D2E-5850-8E2B-274936AE5BCB

Bembidium (Peryphus) chloreum Bates, 1873
Bembidion (Asioperyphus) sapporense Jedlička, 1951 syn. nov.

#### Notes.

The subgenus Politophanes Müller-Morzfeld, 1998 was restored by Schmidt (2018), after Toledano (2008) downgraded *Politophanes* to species group of subgenus Ocydromus Clairville, 1806. The subgenus includes the species that show particular endophallic characters (main sclerite from slightly arcuate to clearly bent, S-shaped, even long and coiled in a ring in the central portion in some species). *Bembidion chloreum*, currently listed as member of subgenus Peryphus, shows endophallic characters that clearly include it in the subgenus Politophanes.

*Bembidion sapporense* Jedlička, 1951, currently assigned to the subgenus Asioperyphus Vysoký, 1986, was described from a single male specimen: we examined the holotype (NMPC) and, based on the aedeagal characters (long main sclerite, coiled in a ring at middle) we assign it to the subgenus Politophanes. The labels of the specimen (Fig. 4) show that our colleague Seiji Morita (Tokyo), a specialist in Japanese Carabidae, particularly in the Bembidiina, identified the specimen as *Bembidion chloreum* Bates in 1991. Later he confirmed this identification and authorized us to state it formally here (Morita, pers. comm.): we are very grateful for that. Therefore we state the following synonymy: Bembidion (Asioperyphus) sapporense Jedlička, 1951 = Bembidion (Politophanes) chloreum Bates, 1873 syn. nov.

### Bembidion (Politophanes) gotoense

Taxon classificationAnimaliaColeopteraCarabidae

Habu, 1973 comb. nov.

27481794-970A-53F7-A289-516E70A74D55

Bembidion (Peryphus) gotoense Habu, 1973

#### Notes.

Habu (1973a) described Bembidion (Peryphus) gotoense from Kyushu, Japan, and in a note reported the closeness of the species to B. (P.) amurense trajectum Netolitzky, 1939 (currently assigned to the subgenus Politophanes Müller-Motzfeld, 1998); *B. gotoense* must therefore also be transferred to *Politophanes*.

### Bembidion (Politophanes) shunichii

Taxon classificationAnimaliaColeopteraCarabidae

Habu, 1973 comb. nov.

25904553-369B-5B0E-9176-71D345D905C5

Bembidion (Peryphus) shunichii Habu, 1973

#### Notes.

Habu (1973b) described Bembidion (Peryphus) shunichii from Formosa [Taiwan] from three females. Later Toledano (2009) assigned this species to the subgenus Ocydromus Clairville, 1806 s. l., “*lenae* Csiki, 1928 group” (currently referred to the subgenus Politophanes Müller-Motzfeld, 1998); the image provided of the aedeagus by Toledano (2009) confirms the attribution to the subgenus Politophanes proposed here.

### Bembidion (Politophanes) umeyai

Taxon classificationAnimaliaColeopteraCarabidae

Habu, 1959 comb. nov.

46890448-841D-5346-B21D-013009A869F5

Bembidion (Peryphus) umeyai Habu, 1959

#### Notes.

Habu (1959) describes Bembidion (Peryphus) umeyai from Hokkaido, Japan, reporting that the species is closely related to Bembidion (Peryphus) amurense trajectum Netolitzky, 1939 (currently listed in the subgenus Politophanes). Later, the species was listed in Marggi et al. (2003) in two different positions, within the subgenus Ocydromus but with the incorrect spelling *umeayi* (sic!) and within *Bembidion* “incertae sedis” with the correct name “*umeyai*”. The same mistake, reported to us by our colleague Katsuyuki Terada (Hiroshima), also occurs in Marggi et al. (2017). In Habu’s (1959) original description and in Morita (2001) the illustration of the aedeagus of *umeyai* shows that the species belongs to the subgenus Politophanes.

### Bembidion (Politophanes) yoshidai

Taxon classificationAnimaliaColeopteraCarabidae

Morita, 2009 comb. nov.

55B03891-2495-580C-9F14-1EF5CF953C0D

Bembidion (Peryphus) yoshidai Morita, 2009

#### Notes.

Morita (2009a) described *Bembidion yoshidai* from SW Japan and in a note reported that the species is closely related to *Bembidion trajectum* Netolitzky, 1939 (currently listed as *Politophanes*); slightly later, Morita himself (2009b) followed Toledano (2008, 2009) and assigned the species to the subgenus Ocydromus Clairville, 1806 s. l. Based on the aedeagal characters reported by Müller-Morzfeld (1998) and Schmidt (2018), *yoshidai* must also be transferred to *Politophanes*.

### Bembidion subg. Pseudosinocys Toledano, 2005

Marggi et al. (2017: 334) reported *Pseudosinyocys* (sic!), while the correct spelling of the subgenus is *Pseudosinocys*.

### Bembidion (Terminophanes) sjoelanderi

Taxon classificationAnimaliaColeopteraCarabidae

Jedlička, 1965 comb. nov.

EA66277F-4A8E-5F6C-BB05-F18EAB7C68CC

Bembidion (Peryphus) sjoelanderi Jedlička, 1965
Bembidion (Terminophanes) pseudoconsumatum Kirschenhofer, 1984 syn. nov.

#### Notes.

Jedlička (1965) described Bembidion (Peryphus) sjoelanderi from China, neglecting to specify the exact locality or mention the number of type specimens. Later, following the further division of the subgenus Peryphus Dejean, 1821 into several separate subgenera, the species has always been listed as *Bembidion* species “incertae sedis” (e.g., Toledano 2000; Marggi et al. 2017). We received a photograph of the holotype, female, of *sjoelanderi*, from NHRS and the examination of some exoskeletal characters (pronotum with laterobasal carina rudimental or absent; pronotum with basal punctation absent or reduced; elytral striae 1, 2, and 5 deeply impressed up to the apex) suggests that the species should be assigned to the subgenus Terminophanes Müller-Motzfeld, 1998. After a further comparison of *sjoelanderi* with many specimens of Bembidion (Terminophanes) pseudoconsumatum Kirschenhofer, 1984, species showing a marked variability in the pronotal width/pronotal length ratio (1.125 to 1.308) we were convinced that the members of both taxa were conspecific, therefore we state the following synonymy: Bembidion (Terminophanes) pseudoconsumatum Kirschenhofer, 1984 = Bembidion (Terminophanes) sjoelanderi Jedlička, 1965 syn. nov.

### Bembidion (Testedium) idriae Meschnigg, 1934

*Bembidion idriae* Meschnigg, 1934 was declared a “species inquirenda” (Neri et al. 2013), and should be added to the species “incertae sedis”.

### Bembidion (Trepanedoris) doris Panzer, 1797

The year of description in Marggi et al. (2017) was mistakenly stated as 1796 and should be corrected to 1797.

### *Ocys tachysoides* Antoine, 1933

In Marggi et al. (2017: 340) the abbreviation GB was typed twice and one should be deleted.

### Sinechostictus (Sinechostictus) cribrum cribrum (du Val, 1851)

The year of description in Marggi et al. (2017) was erroneously stated as 1852 and should be corrected to 1851.

### Sinechostictus (Sinechostictus) dahlii

Taxon classificationAnimaliaColeopteraCarabidae

(Dejean, 1831)

D76351AC-D85D-5BF7-805D-69C377A024B8

Bembidion (Synechostictus) (sic!) dahli ssp. laevigaster De Monte, 1949
Bembidion (Synechostictus) (sic!) dahli ssp. nordafricanum De Monte, 1949

#### Notes.

In Marggi et al. (2017: 341) the taxa *Sinechostictus laevigaster* (De Monte, 1949) and *S. nordafricanus* (De Monte 1949), synonyms of *S. dahlii*, were listed in the same font size as the valid species, instead of the font of the synonyms. The mistake is also recognizable by their order of appearance in the catalogue, in which the species are listed in alphabetical order: *S. laevigaster* and *S. nordafricanus* are listed after *S. dahlii*, but they are followed by *S. decoratus* Duftschmid 1812. After *laevigaster* and *nordafricanus* is mistakenly reported the geographic distribution, which is not reported after the synonyms in the rest of the volume: probably this misleaded the typographers.

Both taxa should be considered synonyms of *S. dahlii*, as stated by Ortuño and Toribio (2005). Moreover, Algeria must be added to the distribution pattern of the species, as reported by De Monte (1949). Current distribution: **E**: FR, IT, SP; **N**: **AG**, LB, MO, TU.

### Sinechostictus (Sinechostictus) ruficornis (Sturm, 1825)

In the revision of the Caucasian species of *ruficorne* – *stomoides* group, Fassati (1992) ascertains that *Sinechostictus ruficornis* (Sturm, 1825) was not present in the Caucasian region; therefore the Russian Caucasus (ST) must be removed from its distribution.

### Sinechostictus (Sinechostictus) multisulcatus

Taxon classificationAnimaliaColeopteraCarabidae

(Reitter, 1890)

D36A7C23-F3A4-57DF-9B76-E3070303984D

Sinechostictus (Sinechostictus) multisulcatus lubricus Lutsnik, 1938
Sinechostictus (Sinechostictus) multisulcatus cariniger Korge, 1971 syn. nov.

#### Notes.

In the revision of the Caucasian species of *ruficorne* – *stomoides* group, after downgrading *S. lubricus* Lutsnik, 1938 and *S. cariniger* Korge, 1971 to subspecies of *S. multisulcatus* Reitter, 1890, Fassati (1992) defined the distribution of the subspecies (*S. multisulcatus multisulcatus* from high altitudes in Central Caucasus; *S. cariniger* from NE Turkey and Georgia; *S. lubricus* from the Western Caucasus, Lesser Caucasus, and Georgia). He also described some diagnostic exoskeletal characters, but in the text frequently repeated the words “in general” and “often”, so that we suppose that, in his opinion, overlapping of characters are frequent in the subspecies. Kryzhanovskij et al. (1995) list the three subspecies from the Caucasus, but a note by Belousov (Kryzhanovskij et al. 1995: 90, note no. 188) says: “… since the infraspecific structure of *multisulcatum* Rtt. remains obscure”. Later *S. lubricus* was considered by all authors as a synonym of *S. multisulcatus* (Lorenz 1998, 2005; Marggi et al. 2003), while *S. cariniger*, formerly listed as synonym of *S. multisulcatus* in Lorenz (1998), was later considered a valid subspecies (Marggi et al. 2003; Marggi et al. 2017).

We examined several specimens from NE Turkey, Georgia, Azerbaijan (new record: N Azerbaijan, Kusary, Shakh-Nabad-Tshai, 3400 m), and southern Russia (Abchasia, Sochi, Krasnodarski Kraj and, confirming *S. lubricus* as a synonym, we think that *S. cariniger* should also be synonymized with the nominotypical form. Therefore we state the following synonymy, with the junior synonym listed first: Sinechostictus (Sinechostictus) multisulcatus cariniger Korge, 1971 = Sinechostictus (Sinechostictus) multisulcatus (Reitter, 1890) syn. nov. Current distribution: **E**: **AB**, GG, ST; **A**: TR.

### Sinechostictus (Sinechostictus) tarsicus

Taxon classificationAnimaliaColeopteraCarabidae

(Peyron, 1858)

B135F7F6-36A3-5705-9BAD-8B9AB3BEE1C5

Sinechostictus (Sinechostictus) effluviorum (Peyron, 1858)

#### Notes.

In Neri et al. (2015)*Sinechostictus tarsicus* (Peyron, 1858) is synonymized with *S. effluviorum* (Peyron, 1858). In Paill et al. (2018: 273), based on a personal communication from Wolfgang Lorenz, the name *tarsicus* was restored (Schaum 1861), as first reviewer, and synonymized *effluviorum* with *tarsicus.* We think that this act is correct, and we confirm that Sinechostictus (Sinechostictus) effluviorum (Peyron, 1858) is a junior synonym of Sinechostictus (Sinechostictus) tarsicus (Peyron, 1858). The mistake was probably due to a misunderstanding of the Schaum’s (1861) considerations. Furthermore, we report the species for Lebanon (Nabeh Safa, 1000 m (PN) and Marjayoun, env. Litani river, 280 m (CR)). Current distribution: **E**: AL, AU, BH, BU, CR, GR, HU, IT, MC, SL, ST, SZ, TR, YU; **A**: CY, IN, IQ, IS, **LE**, SY, TR.

## Errata in references

Page 1318: the reference datum “Sahlberg R.F. 1844: *In faunam insectorum Rossicam symbola*, *novas ad Ochotzk lectas carabicorum species continens*. Acta Societatis Scientiarum Fennicae 3: 1–66” should be replaced by: “*In faunam insectorum Rossicam symbola*, *novas ad Ochotzk lectas carabicorum species continens.* Helsingforsiae: ex off. typ. Frenckelliana, 66 pp.”.

## Supplementary Material

XML Treatment for Asaphidion ganglbaueri

XML Treatment for Bembidion (Euperyphus) dimidiatum

XML Treatment for Bembidion (Peryphanes) fraxator

XML Treatment for Bembidion (Peryphanes) sanatum

XML Treatment for Bembidion (Peryphus) obscurellum

XML Treatment for Bembidion (Peryphus) psuchrum

XML Treatment for Bembidion (Peryphus) subcostatumvau

XML Treatment for Bembidion (Plataphus) pseudolucillum

XML Treatment for Bembidion (Politophanes) chloreum

XML Treatment for Bembidion (Politophanes) gotoense

XML Treatment for Bembidion (Politophanes) shunichii

XML Treatment for Bembidion (Politophanes) umeyai

XML Treatment for Bembidion (Politophanes) yoshidai

XML Treatment for Bembidion (Terminophanes) sjoelanderi

XML Treatment for Sinechostictus (Sinechostictus) dahlii

XML Treatment for Sinechostictus (Sinechostictus) multisulcatus

XML Treatment for Sinechostictus (Sinechostictus) tarsicus
